# Return-to-Work Predictions for Chinese Patients With Occupational Upper Extremity Injury: A Prospective Cohort Study

**DOI:** 10.3389/fmed.2022.805230

**Published:** 2022-07-05

**Authors:** Zhongfei Bai, Jiaqi Zhang, Chaozheng Tang, Lejun Wang, Weili Xia, Qi Qi, Jiani Lu, Yuan Fang, Kenneth N. K. Fong, Wenxin Niu

**Affiliations:** ^1^Shanghai YangZhi Rehabilitation Hospital (Shanghai Sunshine Rehabilitation Centre), School of Medicine, Tongji University, Shanghai, China; ^2^Department of Rehabilitation Sciences, The Hong Kong Polytechnic University, Kowloon, Hong Kong SAR, China; ^3^Capacity Building and Continuing Education Center, National Health Commission of the People's Republic of China, Beijing, China; ^4^Department of Physical Education, Sport and Health Research Center, Tongji University, Shanghai, China

**Keywords:** upper extremity injury, return-to-work, vocational rehabilitation, support vector machine, machine learning, occupational health

## Abstract

**Objective:**

We created predictive models using machine learning algorithms for return-to-work (RTW) in patients with traumatic upper extremity injuries.

**Methods:**

Data were obtained immediately before patient discharge and patients were followed up for 1 year. K-nearest neighbor, logistic regression, support vector machine, and decision tree algorithms were used to create our predictive models for RTW.

**Results:**

In total, 163 patients with traumatic upper extremity injury were enrolled, and 107/163 (65.6%) had successfully returned to work at 1-year of follow-up. The decision tree model had a lower F1-score than any of the other models (t values: 7.93–8.67, *p* < 0.001), while the others had comparable F1-scores. Furthermore, the logistic regression and support vector machine models were significantly superior to the k-nearest neighbors and decision tree models in the area under the receiver operating characteristic curve (t values: 6.64–13.71, *p* < 0.001). Compared with the support vector machine, logistical regression selected only two essential factors, namely, the patient's expectation of RTW and carrying strength at the waist, suggesting its superior efficiency in the prediction of RTW.

**Conclusion:**

Our study demonstrated that high predictability for RTW can be achieved through use of machine learning models, which is helpful development of individualized vocational rehabilitation strategies and relevant policymaking.

## Introduction

Occupational accidents are the most common causes of arm and hand injuries in China. A previous dataset, collected in Chinese cities with concentrated industrial development, showed that 85.4% of patients acquired their injuries in manufacturing industries; severe injuries commonly resulted from working with food, furniture, non-metallic minerals, and wood products (1).

A return-to-work (RTW) is the goal of rehabilitation for patients with work-related injuries. There have been numerous factors for successful RTW in patients with traumatic upper extremity (UE) injury in other countries (2, 3), including sociodemographic factors (e.g., age, educational level, and income), severity/location of injury (e.g., type of injury, joint injury, amputation), and function of the involved UE (e.g., strength, finger dexterity, and participation in purposeful tasks). Although these factors have enriched our understanding of what may influence patient employment after injury, there are two major limitations that should be addressed. First, it is impractical for rehabilitation service providers to collect extensive data from every patient to predict RTW in clinical settings. Therefore, it is important to create predictive models with higher prediction performance using a smaller number of factors. Second, RTW is not a purely biomedical process; on the contrary, many relevant cultural factors may be involved. Over the past decades, although some epidemic studies have reported the prevalence of hand injury and its prognostic factors in China (1), few authors have investigated which factors may contribute to patients' successful RTW or long-term absence from work after a standard rehabilitation program. It may also limit stakeholders in formation of appropriate policies, such as which patients should be endorsed for sick leave extension.

Conventional statistical methods, such as parametric tests of group means, logistical regression, the Kaplan-Meier method and Cox regression analysis, were used to explore and find predictors for RTW. However, the performance of RTW prediction based on predictor thresholds has not been examined in most studies; this could bring into question how the factors can correctly predict RTW in a specific time frame. Machine learning makes classifications and predictions based on probabilistic modeling and has been widely employed to solve industrial problems, such as prediction of project safety performance at construction sites (4). Recently, this approach has attracted researchers' attention in the biomedical and healthcare fields (5), in hopes of predicting brain disorders using neuroimaging data (6) or classifying the risk of developing a sudden illness, such as stroke (7). Lee and Kim (8) created machine learning models to predict RTW for vocational rehabilitation patients injured in an industrial accident; a high prediction performance was found, as indicated by high areas under the receiver operating characteristic (ROC) curves. Machine learning is still a novel approach for vocational rehabilitation, and more research is warranted in additional patients after an occupational accident.

We conducted a prospective cohort study in Shanghai, enrolling patients after traumatic UE injury due to occupational accidents, and all patients were followed up for 1 year. Four commonly examined algorithms, namely, k-nearest neighbors (kNN), logistic regression, support vector machine (SVM), and decision tree, were used to select the factors of importance for RTW. The predictability of the four models was then evaluated.

## Materials and Methods

### Study Design and Participants

This was a prospective cohort study from January 2016 to December 2017, which enrolled patients after traumatic UE injury, admitted to Shanghai YangZhi Rehabilitation Hospital for treatment.

Patients were enrolled in the cohort if they met the following criteria: patients with traumatic UE injury, such as bone fracture and tendon injury; work-related injury identified by the Shanghai Municipal Human Resources and Social Security Bureau; age ≥18 years; first-ever rehabilitation experience after injury. We excluded patients if they met any of the following criteria: comorbid injuries in any other body region or did not complete the rehabilitation. This study was approved by the Research Committee of the Shanghai YangZhi Rehabilitation Hospital (No. YZ2016-097). Written informed consent was obtained from all patients.

### Data Description

Patient demographics, injury information, RTW expectation, physical work demands, functional assessments, and a self-rating scale for the severity of post-traumatic stress disorder (PTSD) were assessed by two occupational therapists before patient discharge. These data, with a total of 27 variables, were further used for machine learning modeling.

Patient demographics included age, sex, marital status, and educational level. For injury information, time since injury in number of days, injured hand dominance (i.e., dominant, non-dominant, or bilateral), and injury location (i.e., finger, wrist, forearm, elbow, upper arm, shoulder, or multiple locations) were collected. The intensity of chronic pain due to injuries was measured using a visual analog scale ranging from zero to ten. Zero indicated no pain at all, while 10 signified pain as bad as possible.

Patients were asked about their expectation of RTW using a 5-point Likert scale ranging from zero to four. One and four represented no expectation and complete expectation, respectively. Likewise, patients' family members were asked to rate the extent to which they expected patients to return to work. If the patients' family members were not reachable, the patients answered this question. We also surveyed employers' attitudes toward RTW because they are crucial. However, employers are not usually reachable, and patients were asked to rate the extent to which their employers expected RTW, based on previous communications.

Physical work demands were classified as sedentary, light, medium, heavy, or very heavy, according to work intensity and frequency. Grip and pinch strength were measured using a Jamar hand dynamometer (9). The EvalTech system (BTE, Hanover, Germany) was used to measure the lifting strength of the bilateral UEs and the carrying strength at the waist and shoulder level. Hand dexterity was quantified by the Purdue Pegboard Test, which involved counting the number of objects inserted during the five subtests (10). The capacity of injured UEs to engage in purposeful and skillful tasks was evaluated using the Chinese version of the Disabilities of the Arm, Shoulder, and Hand (DASH) score (11). The DASH is a self-rated questionnaire that measures the severity of disability and symptomology when performing a given task. The DASH score ranges from 0 to 100, with a higher score indicating a more severe UE disability. The severity of PTSD symptoms was evaluated using the Chinese version of the PTSD Checklist–civilian version (PCL-c), with a higher score indicating more severe symptoms of PTSD (12). All patients were followed-up for 1 year by a social worker via telephone. A successful RTW case was defined as a patient who returned to work for at least one month in the first year after discharge.

### Machine Learning Modeling

In this study, kNN, logistic regression, SVM, and decision tree algorithms were used to train predictive models for the dependent outcome (i.e., RTW at 1-year follow-up), which was defined as binary. Univariant logistic regression tests indicated that 17/27 variables (Table 1) were significantly predictive of RTW; these were then selected as input variables for model training. In view of the small sample size (*n* = 163), overfitting could be easily induced, regardless of the algorithm, if a large number of variables were input. Therefore, we further selected the best subsets of variables for kNN, logistic regression, and SVM using an exhaustive feature search. Specifically, the variable number of subsets started from one and all possible subsets with one variable were created. Then, the models were trained with all subsets, and the one with the most optimal performance was selected. Finally, the variable number of subsets was increased, and the optimal subset updated. The search was stopped if the performance of the models did not improve, even as more variables were input. The aforementioned search was not applied for decision tree model training because this algorithm can select the most relevant variables automatically, according to their importance, and discard irrelevant variables.

**Table 1:** Univariant logistic regression result comparison between RTW and non-RTW patients.

**Variables**	**RTW (*n* = 107)**	**Non-RTW (*n* = 56)**	***t*, Mann-Whitney, χ^2^**		**Univariant logistic regression**	
			**Statistic**	**p**	**OR**	**p**
Age (years)	37.4 ± 9.7	39.3 ± 10.9	1.12	0.265	0.982	0.263
Sex						
Male	79	38	0.65	0.421	0.748	0.422
Female	28	18				
Marital status						
Married	90	47	<0.01	1.000	0.986	0.976
Single	17	9				
Educational level						
Illiteracy	1	3	−2.58	0.010	1.713	0.007
Primary school	10	8				
Junior middle school	47	29				
High middle school	35	14				
College diploma or higher	14	2				
Time since injury (days)	142.1 ± 76.4	172.9 ± 91.4	2.28	0.024	0.996	0.029
Injured hand dominance						
Dominant	53	27	1.60	0.512	0.845	0.553
Non-dominant	51	25				
Bilateral	3	4				
Injury location						
Finger	67	21	11.50	0.057	0.813	0.025
Wrist	18	14				
Forearm	5	7				
Elbow	5	4				
Upper arm	2	2				
Shoulder	8	5				
Multi-location	2	3				
Pain intensity	3.0 ± 2.0	3.2 ± 2.2	0.70	0.486	0.946	0.484
Patient's expectation of RTW	2.6 ± 1.0	2.0 ± 1.1	−3.17	0.002	1.661	0.001
Family's expectation of RTW	2.6 ± 1.0	2.0 ± 1.2	−2.84	0.005	1.647	0.002
Employer's expectation of RTW	2.5 ± 0.9	2.0 ± 0.9	−3.26	0.001	1.909	0.001
Physical work demands						
Sedentary	1	0	−0.35	0.724	0.947	0.741
Light	21	12				
Medium	40	17				
Heavy	27	18				
Very heavy	18	9				
Grip strength of the injured UE (kg)	10.2 ± 8.9	17.8 ± 12.0	−4.56	<0.001	1.072	<0.001
Grip strength of the healthy UE (kg)	36.2 ± 10.5	33.2 ± 10.5	−1.72	0.087	1.027	0.088
Pinch strength of the injured UE (kg)	5.7 ± 3.2	3.7 ± 2.8	−3.79	<0.001	1.236	<0.001
Pinch strength of the healthy UE (kg)	10.1 ± 4.7	9.3 ± 4.4	−1.08	0.161	1.047	0.289
Lifting strength of the injured UE (kg)	27.3 ± 16.8	17.0 ± 12.6	−4.06	<0.001	1.055	<0.001
Lifting strength of the healthy UE (kg)	47.8 ± 18.9	42.1 ± 17.6	−1.88	0.062	1.017	0.065
Carrying strength at waist (kg)	27.0 ± 12.7	16.3 ± 12.0	−5.20	<0.001	1.075	<0.001
Carrying strength at shoulder (kg)	21.8 ± 11.3	12.5 ± 9.2	−5.30	<0.001	1.094	<0.001
Purdue pegboard test						
Injured hand	12.2 ± 4.2	9.5 ± 5.4	−3.36	0.001	1.128	0.001
Healthy hand	16.2 ± 1.8	15.7 ± 2.1	−1.57	0.119	1.150	0.120
Both hands	11.2 ± 4.2	8.3 ± 4.7	−3.98	<0.001	1.169	<0.001
Injured + healthy + both	39.6 ± 8.6	33.5 ± 10.9	−3.67	<0.001	1.069	<0.001
Assembly	28.2 ± 10.3	22.5 ± 12.7	−2.90	0.005	1.045	0.003
DASH	34.5 ± 19.3	43.8 ± 17.3	3.00	0.003	0.974	0.004
PCL-c	35.4 ± 12.7	39.8 ± 13.9	2.03	0.044	0.975	0.047

*All variables were compared between patients who returned to work and those who did not. Independent sample t-tests (t) were used for continuous data, while Mann-Whitney tests were used for ordinal data. The differences on categorical data were checked by using Chi-square tests (χ^2^). In addition, univariant logistic regression tests were employed to investigate whether variables were individually predictive for RTW. Only those variables which showed significant predictability were included for machine learning modeling. In this table, continuous data are expressed as mean ± SD, ordinal and nominal data are expressed as a number. RTW, return to work; OR, odds ratio; UE, upper extremity; DASH, Disability of the Arm, Shoulder and Hand; PCL-c, Post-traumatic Stress Disorder Checklist–civilian version*.

In the validation method, data were separated into two datasets for model training (70%) and validation (30%). Because of the limited sample size, random separation could produce substantially varied and unreliable model performance. Therefore, each model was trained 100 times to obtain its performance distribution, which was then compared among the models. The F1-score, which is the harmonic mean of precision and recall, was used to evaluate the performance of models on validation datasets. This was done even with the imbalance of outcome classes, due to 65.6% of our included patients successfully RTW. Optimal hyperparameter combinations were selected using a grid search method. The scikit-learn toolkit (version 0.24.0) was used for model training and validation (13).

### Statistical Analysis

Statistical analysis was performed using SPSS22 (IBM, NY, and USA) with a level of significance of 0.05. Initially, the baseline differences between RTW and non-RTW patients were compared using independent *t*-tests, Mann-Whitney tests, or chi-square tests when appropriate. Second, univariate logistic regression was used to determine whether individual variables were predictive of RTW. Third, to evaluate performance of the four models, F1-scores and areas under the ROC were compared using one-way repeated measures analysis of variance (ANOVA), and *post-hoc* analyses were conducted using paired *t-*tests with the Bonferroni correction (corrected alpha threshold = 0.05/6). One-way ANOVA was used to examine whether the F1 score from 100 training sessions was significantly different from sets with larger numbers of training sessions.

## Results

**Figure 1:**
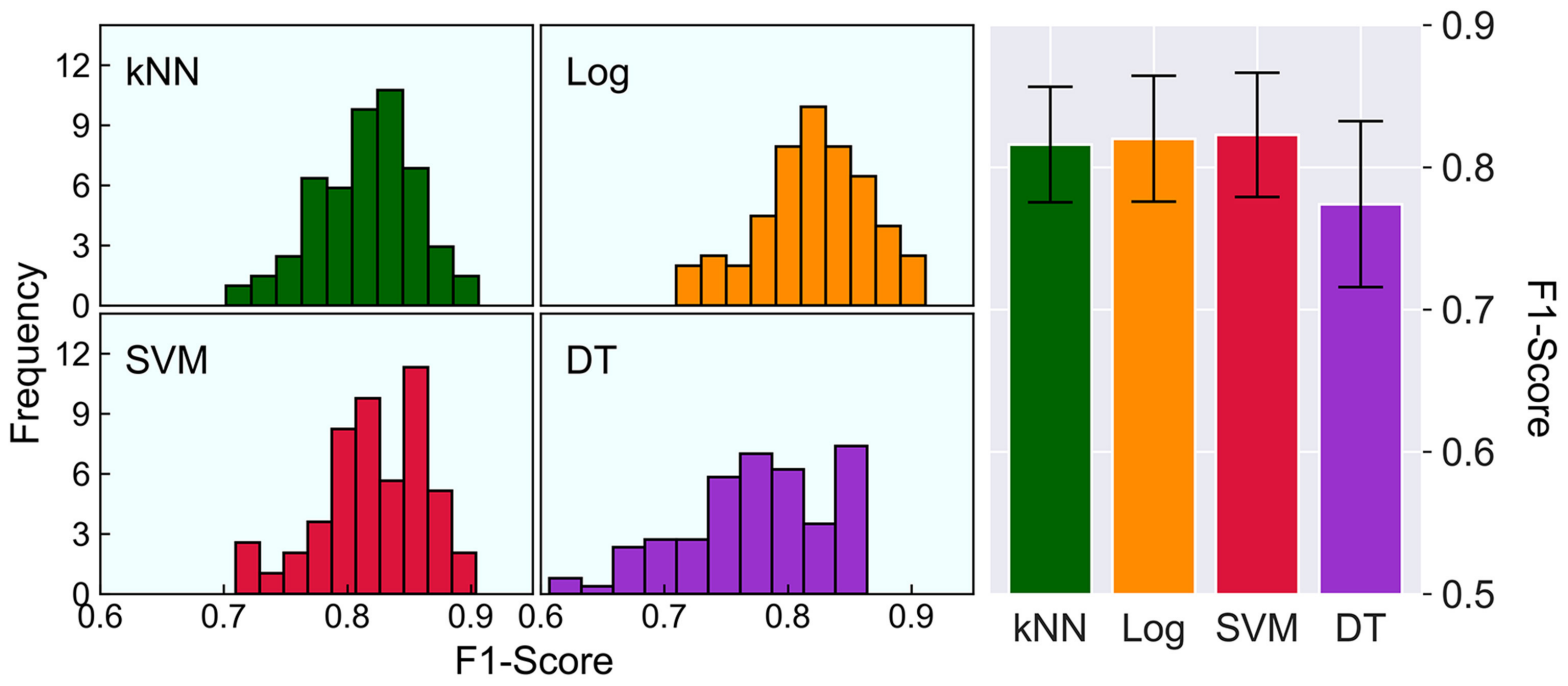
Comparison on F1-scores of the four models. The left histograms show the distribution of the F1-score, and the right bar chart shows a direct comparison on the F1-scores of kNN (0.816 ± 0.041), Log (0.820 ± 0.044), SVM (0.823 ± 0.044) and DT (0.774 ± 0.059). The error bars represent one standard deviation of uncertainty. kNN, k-nearest neighbors; Log, logistic regression; SVM, support vector machine; DT, decision tree.

A total of 179 adult inpatients with traumatic UE injury were enrolled. Ultimately, 163 patients were successfully followed up, of which 107 (65.6%) successfully returned to work by 1-year. Comparisons between RTW and non-RTW patients indicated significant differences in many variables that were also predictive of RTW (Table 1). A one-way repeated measures ANOVA indicated a significant difference in the F1-score among the four models (F = 47.61, *p* < 0.001), as shown in Figure 1. *Post-hoc* analysis by paired *t*-tests found that the decision tree model had a lower F1-score than any of the others (t values ranging from 7.93 to 8.67, all *p* < 0.001, survived Bonferroni correction), and the rest of the comparisons were not significant (t values ranging from 0.92 to 1.73*, p*-values ranging from 0.087 to 0.361). In terms of the factors selected for modeling, time since injury, carrying strength at the waist, carrying strength at the shoulder, Purdue pegboard test score (injured hand), and Purdue pegboard test score (both hands) were five optimal variables for kNN, two variables (patient's expectation of RTW and carrying strength at the waist) for logistic regression, and four [injury located at fingers, patient's expectation to RTW, carrying strength to shoulder, and Purdue pegboard test score (both hands)] for SVM.

**Figure 2:**
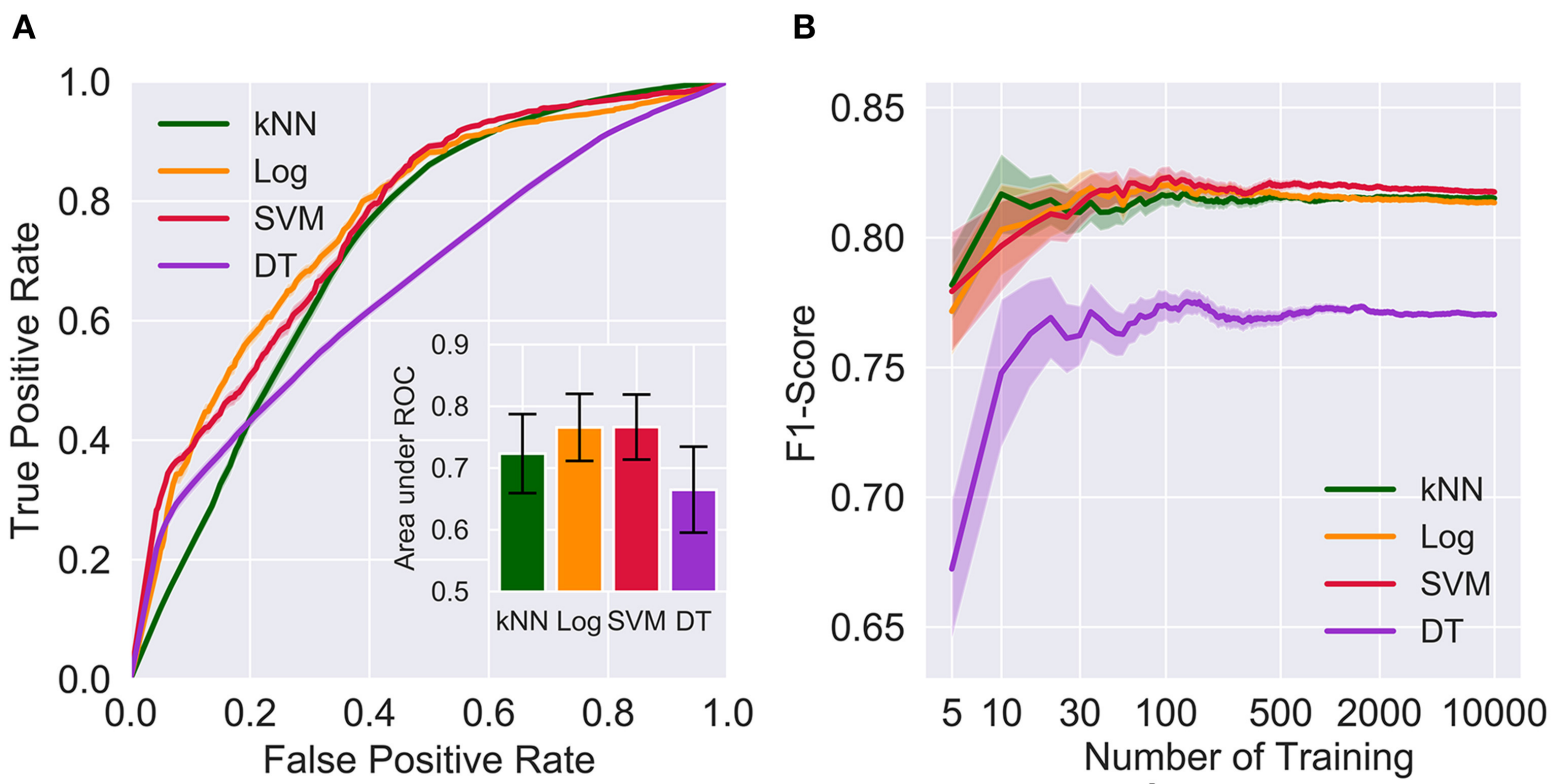
Comparison on the areas under the ROC of the kNN (0.723 ± 0.064), Log (0.766 ± 0.054), SVM (0.766 ± 0.053) and DT (0.665 ± 0.070) and the effects of the number of trainings on F1-scores. The error bars in **(A)** represent one standard deviation of uncertainty. The shaded regions in **(A,B)** represent one standard error of the mean. kNN, k-nearest neighbors; Log, logistic regression; SVM, support vector machine; DT, decision tree; ROC, receiver operating characteristic curve.

The ROC analysis results are shown in Figure 2. One-way repeated measures ANOVA indicated significant differences among the four models (F = 95.48, *p* < 0.001). *Post-hoc* analysis indicated that the logistic regression and SVM models had comparable areas under the ROC (t = 0.13, *p* = 0.896) and were significantly superior to the kNN and decision tree models (t values ranging from 6.64–13.71, all *p* < 0.001, survived Bonferroni correction). In addition, the area under the ROC curve of the kNN model was also significantly larger than that of the decision tree model (t = 6.70, *p* < 0.001, surviving Bonferroni correction).

In view of limited computational resources, each model was trained 100 times. To evaluate the effect of the number of training sessions on performance estimation, number of training sessions was manipulated from 5 to 10,000. As shown in Figure 2B, the F1-score was relatively precise when larger numbers of training (e.g., 500, 2,000, and 10,000) were applied, regardless of the algorithms. In contrast, small numbers of training sessions (e.g., 5, 10, and 30) yielded substantially variable and much lower F1-socres than larger training sets. One-way ANOVA suggested that F1-scores resulting from 100 training sessions were not significantly different from 500, 2,000 or 10,000 training sessions for the kNN (F = 0.110, *p* = 0.954), logistical regression (F = 1.88, *p* = 0.131), SVM (F = 1.95, *p* = 0.119), and decision tree (F = 0.285, *p* = 0.836) models, indicating that 100 times was sufficient for model training (Figure 2).

## Discussion

We demonstrate that machine learning models can be used for RTW prediction in Chinese patients after traumatic UE injuries, indicating high predictive performance. Although both logistical regression and SVM displayed better performance than the others, logistical regression required a smaller number of factors, suggesting its high efficiency. We also discovered a large number of factors which were in line with previous studies associated with RTW (2, 14). Our machine learning models selected several important factors, such as carrying strength at the waist, patient's expectation of RTW, and Purdue pegboard test score (both hands).

RTW factors following various work-related injuries have been analyzed using traditional statistical methods (2, 3, 15). Our study is the first to use machine learning models to predict RTW in patients after a traumatic UE injury. Logistical regression and SVM were the two best algorithms for predicting RTW. Recently, prediction of risk level classification, differential diagnoses, and prognoses of various diseases have been investigated using machine learning models with excellent performance (6, 7, 16). In particular, SVM has shown superior performance (6, 8), which is in line with our findings. While the black-box problem of SVM is a complex mathematical formulation, it is difficult to interpret the model. Most recently, Rudin (17) argued that when addressing practical problems, designing inherently interpretable models is the way forward, rather than trying to explain black box models. By contrast, logistical regression classifies samples based on probability which is easily interpreted. Although comparable performance was obtained with SVM and logistic regression, logistic regression required only two factors, namely, the patient's expectation of RTW and carrying strength at the waist, suggesting its superior efficiency.

Shi et al. (2) reported that the severity of injury as well as pre-injury income were consistent factors for RTW. Recently, Marom et al. reported additional factors contributing to RTW, such as compensation, educational level, self-efficacy, work demands, pain, and physical capacity (3). In our study, pre-injury income was not included because most patients refused to disclose their financial status. Instead of assessing the severity of the injury, a series of functional assessments were conducted for three reasons. First, the initial severity of hand injury is only partially correlated with functional performance, which is more relevant to the probability of RTW after injury (18). Second, our patients had different single or multiple locations of injury, and it was difficult to evaluate the severity using a uniform score. Third, this study was conducted in a rehabilitation hospital and functional assessments were of practical convenience. Among these functional assessments, carrying strength using both hands was an important factor for RTW. A possible explanation might be that most of our patients were manual workers from manufacturing industries, for whom carrying strength is an essential demand to return to previous work (19). In addition, the patient's expectation of RTW was a critical factor selected by both logical regression and SVM. These findings were in line with those by Heijbel et al. (20) that individuals with expectations of RTW had an approximately eight times higher possibility of RTW than those without that expectation.

The main goal of rehabilitation for occupational injuries is to improve overall functional capacity and ultimately facilitate RTW. Accurate prediction of RTW is helpful for individualized vocational rehabilitation treatment plans. For instance, work-hardening training is crucial for patients who have a high probability of returning to previous work; in contrast, patients who are not likely to return to work, due to severe functional impairments, have to seek supported employment, duty modification, or job transition assistance (21, 22). Most recently, Lee and Kim (8) used similar algorithms to predict whether patients could RTW successfully after an industrial accident. Specific assessments of body function were missing in their study. We focused only on patients with traumatic UE injuries; in particular, a series of functional assessments for UEs were included for modeling, making our findings more specific to the targeted population.

We provide a novel direction for stakeholders when formulating policies relevant to occupational RTW. An RTW policy is designed to help injured workers to return to work in a safe and timely manner, which is beneficial for both employers and the workers themselves. Our machine learning models can obtain a patients' probability of RTW based on this previous dataset. Therefore, stakeholders can assign more individualized policies to workers after an injury. Currently, all occupational injury workers identified by the Shanghai Municipal Human Resources and Social Security Bureau can be approved for one-year sick leave with compensation. However, this policy may not be appropriate without consideration of individual body function. For instance, those with worse body function usually have a lower probability of RTW and should be endorsed for sick leave extensions. However, a shorter period was adequate for those with a higher probability of RTW.

This study has some limitations. First, our sample size was small, which may lead to overfitting, even though some modeling strategies have been employed to compensate for this disadvantage. Second, expectations of RTW were assessed using a 5-point Likert scale, which may not be adequate to represent the full construct of expectation. More standardized assessments with better construct validity are recommended for use in future studies, such as the questionnaire used by Sampere et al. (23). Third, only four commonly used machine learning algorithms were investigated, and higher predictability may have been yielded by others.

## Conclusion

RTW can be highly predicted by machine learning models, of which both logistic regression and SVM demonstrated high predictability. In particular, logistical regression selected for only two essential factors: a patient's expectation of RTW and carrying strength at the waist. The selected factors can be considered the most relevant factors for prediction of RTW after traumatic UE injury. Predictive models could contribute to the development of tailor-made vocational rehabilitation programs. Furthermore, machine-learning-based predictive models provide a novel direction for stakeholders while formulating policies relevant to occupational RTW.

## Data Availability Statement

The original contributions presented in the study are included in the article, and further inquiries can be directed to the corresponding author.

## Ethics Statement

The studies involving human participants were reviewed and approved by Research Committee of Shanghai YangZhi Rehabilitation Hospital. All procedures were in accordance with the ethical standards of the responsible committee on human experimentation (institutional and national) and the Helsinki Declaration. The patients/participants provided their written informed consent to participate in this study.

## Author Contributions

ZB, JZ, KF, and WN contributed to conception and design of the study. WX, JL, and YF collected the data. ZB, LW, and QQ organized the algorithms and database. JZ and QQ performed the statistical analysis. ZB wrote the first draft of the manuscript. JZ, CT, LW, and WX wrote sections of the manuscript. All authors contributed to manuscript revision, read, and approved the submitted version.

## Funding

This work was supported by the National Natural Science Foundation of China (32071308), Shanghai Sailing Program (20YF1445100), Shanghai Municipal Science and Technology Major Project (2021SHZDZX0100), and Fundamental Research Funds for the Central Universities.

## Conflict of Interest

The authors declare that the research was conducted in the absence of any commercial or financial relationships that could be construed as a potential conflict of interest.

## Publisher's Note

All claims expressed in this article are solely those of the authors and do not necessarily represent those of their affiliated organizations, or those of the publisher, the editors and the reviewers. Any product that may be evaluated in this article, or claim that may be made by its manufacturer, is not guaranteed or endorsed by the publisher.
